# Repeat workers' compensation claims: risk factors, costs and work disability

**DOI:** 10.1186/1471-2458-11-492

**Published:** 2011-06-22

**Authors:** Rasa Ruseckaite, Alex Collie

**Affiliations:** 1 Institute for Safety, Compensation and Recovery Research, Monash University, Melbourne, Victoria, Australia; 2 Department of Epidemiology and Preventive Medicine, Monash University, Melbourne, Victoria, Australia

## Abstract

**Background:**

The objective of our study was to describe factors associated with repeat workers' compensation claims and to compare the work disability arising in workers with single and multiple compensation claims.

**Methods:**

All initial injury claims lodged by persons of working age during a five year period (1996 to 2000) and any repeat claims were extracted from workers' compensation administrative data in the state of Victoria, Australia. Groups of workers with single and multiple claims were identified. Descriptive analysis of claims by affliction, bodily location, industry segment, occupation, employer and workplace was undertaken. Survival analysis determined the impact of these variables on the time between the claims. The economic impact and duration of work incapacity associated with initial and repeat claims was compared between groups.

**Results:**

37% of persons with an initial claim lodged a second claim. This group contained a significantly greater proportion of males, were younger and more likely to be employed in manual occupations and high-risk industries than those with single claims. 78% of repeat claims were for a second injury. Duration between the claims was shortest when the working conditions had not changed. The initial claims of repeat claimants resulted in significantly (*p < 0.001*) lower costs and work disability than the repeat claims.

**Conclusions:**

A substantial proportion of injured workers experience a second occupational injury or disease. These workers pose a greater economic burden than those with single claims, and also experience a substantially greater cumulative period of work disability. There is potential to reduce the social, health and economic burden of workplace injury by enacting prevention programs targeted at these workers.

## Background

It is common practice for workers' compensation authorities to publish reports on the characteristics of accepted claims, return to work outcomes and client satisfaction[1]. In Australia there is a regular benchmarking report produced by the national co-ordinating authority comparing outcomes (eg benefits paid, pre-injury positions, return to work provisions, dispute resolution, access to common law) between workers' compensation jurisdictions[2]. There have also been a number of reports examining factors associated with workers compensation claim behaviour [3-7]. These reports have identified a number of factors associated with an increased likelihood of an accepted workers' compensation claim, including male gender, younger age and lack of work experience. Those working in certain industries such as manufacturing, and certain occupations such as labouring and trades (requiring physical exertion and extensive manual activity over extended periods) are at greater risk of lodging a workers' compensation claim[8,9]. Similarly, the majority of claims arise from musculoskeletal injury [6,9,10]. Government occupational health and safety (OHS) initiatives are strongly influenced by trends emerging from workers' compensation data, particularly in those jurisdictions where the OHS and workers' compensation activities are managed by a single regulatory authority [5].

Knowledge of risk factors for work-related injury is almost exclusively drawn from analyses that treat each workers' compensation claim as a discrete event. A different approach would be to examine risk of injury within an individual worker over a defined period of time. There is very little published information examining the characteristics of those workers who file repeat workers' compensation claims. Lipscomb and colleagues[11] reported that recurrent back injuries resulting in workers' compensation claims occurred at a rate 80% higher than initial injuries among carpenters. Distributions of first and second back injuries were similar in terms of mechanism and nature of injury. In perhaps the most comprehensive report to date, Cherry and colleagues[12] described the characteristics of Canadian workers' compensation claimants from the province of Alberta who had filed second claims. A reduced time to file a second claim was associated with male gender, younger age and some types of injury or accident. Manufacturing industry and machining trades were at highest risk of an early second claim. Currently, there is a very limited understanding of this phenomenon across a broad range of injuries, industries and occupations. For example, there have been no reports of the circumstances under which a second claim occurs with the same or different employer, in the same or different occupation and industry, or as a consequence of the same or different mechanism of type of injury. Similarly, we are unaware of any reports comparing the work disability or costs arising from second claims to that arising from initial claims.

In the current study we report the data on initial and repeat workers' compensation claims extracted from the compensation system database of WorkSafe Victoria in Australia. This study sought to describe factors associated with repeat workers' compensation claims and to compare the work disability arising in two groups of workers, being (a) those with a single compensation claim during the study period, and (b) those with two workers' compensation claims during the study period.

## Methods

### Workers' Compensation System in Victoria

In the state of Victoria, employers are required to maintain workers' compensation insurance through the Victorian WorkCover Authority (VWA) unless they are able to self-insure or obtain insurance through the national workers' compensation scheme. Approximately 85% of workers in the state are insured by the VWA. A workers' compensation claim may be lodged once the employee has been off work 10 days or when a threshold level of medical expenses has been reached. This amount varied from $407 in 1996 to $582 in 2009. Date of onset is defined as either: (a) the date on which the occupational injury occurs, or (b) for occupational diseases, the date on which a claim was lodged with the VWA. The VWA regulates the workers' compensation system in Victoria, with compensation claims managed by a number of private insurance organisations. Case-level data is collected by the private insurers and provided to the regulator on an on-going basis for the purpose of managing and analysing the performance of the workers' compensation scheme.

### Compensation Research Database

Workers' compensation administrative claims data from 1 January 1986 through to 31 December 2009 were obtained from the VWA. The database contains information necessary for the management of the workers' compensation scheme, including industry, occupation, employer, workplace, demographic, injury, claim cost and payment data. Data were stripped of identifying information including claims numbers, names and contact details of claimants prior to being provided to the research team. Non-identifying 'dummy' ID numbers were assigned to each claim in the database and to each claimant (person). This information was used to identify claimants with single and repeat claims. Institutional ethics approval was gained from Monash University Human Research Ethics Committee for use and disclosure of the claims information.

Industry group data was coded using the VWA defined Workplace Industry Code (WIC). For the purposes of analyses, the WIC codes were converted to unit group (4 digits) Australian New Zealand Standard Industry Classification (ANZSIC 2006) codes using a conversion algorithm developed by the VWA. Occupation group data was collected using the Australian Standard Classification of Occupations (ASCO) version 1 (prior to 1997) or version 2 (from 1997). All data in the database were reported in ASCO v2 (converting, where necessary, from ASCO v1 using standard Australian Bureau of Statistics (ABS) conversion algorithms). For the purposes of analyses, the ASCO v2 codes were then converted to unit group (4 digits) Australian and New Zealand Standard Classification of Occupation (ANZSCO) codes using conversion tables developed by the ABS and incorporating 2006 Australian census data.

Mechanism of injury and bodily location of injury were coded using the Australian standard Type of Occurrence Classification System (TOOCS, 3^rd ^edition) for workplace injury and disease recording[13], and are reported using this classification system. Nature of injury or disease was recorded using the VWA coding system, which was mapped to the TOOCS nature of affliction coding scheme for analysis purposes. Mechanism of injury, bodily location, nature of affliction, occupation and industry were coded by the VWA and the codes were obtained together with the database.

The total cost per claim, total cost of weekly compensation and total costs of medical services were also calculated and converted to 2009 Australian dollar equivalent values. Number of days' income replacement paid (as an indicator of duration of work disability) was also extracted from the database.

### Participants

The sample was defined as persons of working age (≥ 15 years) whose initial accepted claim was lodged during the 5 year period 1^st ^January 1996 to the 31^st ^December 2000. This timeframe was chosen because there were no significant changes to the workers' compensation legislation during the period. Repeat claims occurring up to 31^st ^December 2009 were included in the dataset. We restricted initial claims to those resulting from occupational injury and excluded initial claims occurring for occupational disease, as the compensation data did not allow us to determine the date of onset of the disease. However, repeat claims for both injury and disease were included as this allowed calculation of the probability of an injury claim being followed by second injury claim or a subsequent disease claim. The resulting sample was stratified into two groups, being (1) workers with single claims (group 1); and (2) workers with more than one claim (group 2). All participants with two and more claims were assigned to the second group; however only data from the first two claims was analysed. Most of the claims were closed, however we also included claims that were still open in the current analysis.

### Data analysis

Descriptive analyses were undertaken including a summary of participants in each group by gender and age, occupation and industry. Comparisons between group 1 and group 2 on each of these factors were conducted using Chi-square test. To correct for multiple comparisons, a result was considered significant if the p value was below 0.01. Most analyses focused on the second group, to determine if the second claim (a) arose from occupational injury or disease; (b) arose from the same or different affliction and in the same or different bodily location; (c) occurred while working in the same or different industry and the same or different occupation; (d) occurred while working at the same or different workplace and for the same or different employer. In each case, the number and percentage of participants whose second claim occurred in the same/different categories was calculated, as was the time (in days) between lodgement of the initial and repeat claims. A survival analysis, using a Cox proportional-hazards model, was carried out to estimate the impact of these different variables on time between initial and repeat claims. The time to second claim was calculated from the date the first claim was lodged. In all cases the reference condition for the survival analysis was the condition where no change had occurred between initial and repeat claims (i.e., same employer, same workplace). Finally, for both groups we calculated measures of claim outcome, including total cost per claim, total cost of weekly compensation (i.e., income replacement benefits), total costs of medical services and number of days of income replacement paid. We used t-tests to compare these outcomes in three conditions: (a) group 1 initial claim vs group 2 initial claim; (b) group 1 initial claim vs. group 2 repeat claim; and (c) group 2 initial claim vs. group 2 repeat claim. All analyses were performed using SPSS 17.0.

## Results

### Study sample

A total of 188,402 injured persons (claimants) lodged an initial claim with the VWA during the study period. The majority (N = 170,148 or 90.3%) of these claims were due to occupational injury and were included in the current analyses. Of these, 63% lodged a single claim (Group 1) while the remaining 37% lodged more than one claim (Group 2). Characteristics of both groups are displayed in Table 1. Group 2 was significantly younger and contained a significantly higher proportion of males than Group 1. The most common occupation amongst claimants in both groups was that of labourer and the most common industry was manufacturing. Group 2 comprised a significantly greater proportion of participants working in manual occupations (labourers, technicians and trade workers, machinery operators and drivers) than Group 1, and a significantly smaller proportion of 'white collar' occupations (managers, professionals, clerical and administrative workers, sales workers) than Group 1. Similarly, Group 2 comprised a significantly greater proportion of manufacturing and transport workers than Group 1, and a significantly smaller proportion of workers in other industries including agriculture, retail trade, accommodation and food services, finance, professional and scientific, education, public administration and the arts and recreation services. Among participants in Group 2, 78.8% of repeat claims were for a second occupational injury with the remaining 21.2% being for occupational disease.

**Table 1:** Characteristics of participants with a single claim (Group 1) and multiple claims (Group 2).

Category	Group 1	Group 2
	Single claim	Multiple claims
N (%) participants	107,388 (63%)	62,760 (37%)
Gender *		
N (%) Female	38,432 (35.8%)	17,523 (27.9%)
N (%) Male	62,880 (58.6%)	42,877 (68.3%)
N (%) missing #	6,076 (5.7%)	2,360 (3.8%)
Mean (SD) age in years*	35.1 (12.3)	34.5 (11.4)
Occupation (ANZSCO) *		
Managers	5,387 (5.0%)	1,918 (3.1%)
Professionals	15,564 (14.5%)	7,232 (11.5%)
Technicians & trade workers	20,552 (19.1%)	14,356 (22.9%)
Community & personal service workers	7,463 (6.9%)	5,402 (8.6%)
Clerical & administrative workers	6,238 (5.8%)	1,952 (3.1%)
Sales workers	5,293 (4.9%)	1,732 (2.8%)
Machinery operators & drivers	10,096 (9.4%)	7,385 (11.8%)
Labourers	30,664 (28.6%)	20,362 (32.4%)
Missing #	6,131 (5.7%)	2,421 (3.9%)
Industry (ANZSIC)*		
Agriculture, forestry &fishing	2,864 (2.7%)	1,142 (1.8%)
Mining	314 (0.3%)	232 (0.4%)
Manufacturing	23,519 (21.9%)	18,416 (29.3%)
Electricity, gas, water & waste services	514 (0.5%)	318 (0.6%)
Construction	7,166 (6.7%)	4,315 (6.9%)
Wholesale trade	7.338 (6.8%)	4,140 (6.6%)
Retail trade	9,611 (8.9%)	3.886 (6.2%)
Accommodation & food services	4,139 (3.9%)	1,488 (2.4%)
Transport, postal & warehousing	4,980 (4.6%)	3,580 (5.7%)
Information media & telecommunications	178 (0.2%)	95 (0.2%)
Financial and insurance services	966 (0.9%)	216 (0.3%)
Rental, hiring & real estate services	716 (0.7%)	361 (0.6%)
Professional, scientific & technical	2,775 (2.6%)	807 (1.3%)
services	7,277 (6.8%)	4,413 (7.0%)
Administrative & public support services	1,790 (1.7%)	879 (1.4%)
Public administration & safety	7,422 (6.9%)	3,435 (5.5%)
Education & training	12,180 (11.3%)	7,043 (11.2%)
Health care & social assistance	3,381 (3.1%)	1,802 (2.9%)
Arts & recreation services	2,947 (2.7%)	2,974 (4.7%)
Other services	7,311 (8.4%)	3,188 (5.1%)
Missing #		
Second claim		
N (%) occupational injury	-	49,474 (78.8%)
N (%) occupational disease	-	13,286 (21.2%)

# Missing category denotes data that was not recorded in the database.

*p < 0.001

### Nature of affliction and bodily location

Nearly half (45.5%) of all repeat claims resulted from a different affliction to a different bodily location than the initial claim (Table 2). The remaining repeat claims were distributed across the three other categories. The mean number of days between initial and repeat claims was lowest in those participants whose second claim was for the same affliction occurring in the same bodily location, followed by those with the same affliction in a different bodily location and those with a different affliction. Means, standard deviations (SD) and Hazard Ratios (HR) with 95% confidence intervals (CIs) representing the risk of a rapid second claim relative to the reference condition are shown in Table 2. The risk of a more rapid repeat claim decreased when the bodily location and/or nature of affliction changed compared to the reference condition. This was most evident when the nature of the affliction changed. Table 3 illustrates nature of affliction of the initial claim in both claimant groups.

**Table 2:** Characterisation of repeat claims by nature of affliction and bodily location categories.

Category*	N (%) of participants	**Mean ± SD days between 1**^**st **^**and 2**^**nd **^**claims**	HR [95% CI]
Same affliction/Same bodily location *(Reference condition)*	11,586 (18.5%)	725 ± 807	1
Same affliction/Different bodily location	12,658 (20.2%)	866 ± 893	.81 [.79 - .83]
Different affliction/Same bodily location	9,907 (15.8%)	1,331 ± 1,143	.55 [.53 - .57]
Different affliction/Different bodily location	28,609 (45.5%)	1,248 ± 1,123	.59 [.58 - .61]
Total	62,760	1,088 ± 1,057	

Mean ± SD days represents average time between the dates of claims lodged. Estimated Hazard Ratio (HR) from Cox regression with 95% Confidence intervals (CI) is shown in the right-most column.

* Nature of affliction and bodily location categories were coded using the standard Type of Occupational Occurrence Classification System (TOOCS).

**Table 3:** Nature of affliction at time of first claim for single and repeat claimant groups.

Nature of Affliction	Group 1 (N = 107,388)	Group 2 (N = 62,760)
Intracranial injuries	609 (0.6%)	353 (0.6%)
Fractures	7,587 (7.1%)	3,094 (4.9%)
Wounds, Lacerations, Amputations & Internal Organ Damage	34,385 (32.0%)	20,082 (32.0%)
Burns	2,807 (2.6%)	1,629 (2.6%)
Injury to Nerves & Spinal Cord	61 (0.1%)	23 (0.0%)
Traumatic Joint/Ligament & Muscle/Tendon Injury	57,407 (53.5%)	34,849 (55.5%)
Other Injuries	4,532 (4.3%)	2,730 (4.4%)

### Occupation and industry

Nearly three quarters (74.8%) of repeat claims arose from workers who were engaged in the same industry as during their initial claim, however over a third (36.3%) had changed occupations between initial and repeat claims. The risk of a more rapid repeat claim decreased in those workers who had changed industries between initial and repeat claims and this was most evident when both the industry and occupation had changed (Table 4).

**Table 4:** Characterisation of repeat claims by occupation and industry categories.

Category*	N (%) of participants	**Mean ± SD days between 1**^**st **^**and 2**^**nd **^**claims**	HR [95% CI]
Same industry/Same occupation *(Reference condition)*	24,168 (38.5%)	927 ± 964	1
Same industry/Different occupation	22,794 (36.3%)	925 ± 949	.86 [.85 - .88]
Different industry/Same occupation	4,474 (7.1%)	1,662 ± 1,149	.94 [.91 - .97]
Different industry/Different occupation	8,696 (13.9%)	1,764 ± 1,166	.82 [.80 - .85]
Missing	2,628 (4.2%)		
Total	62,760	1,088 ± 1,057	

Mean ± SD days represents average time between the dates of claims lodged. Estimated Hazard Ratio (HR) from Cox regression with 95% Confidence intervals (CI) is shown in the right-most column.

* Occupation was coded using the Australian New Zealand Standard Classification of Occupations (ANZSCO). Industry was coded using the Australian New Zealand Standard Industry Classification (ANZSIC) coding system.

### Employer and workplace location

Slightly more than half (55.2%) of workers' filing repeat claims did so while working for the same employer at the same workplace location as during their initial claim. One in ten remained employed by the same employer but had changed workplace locations, while the remaining third (34.4%) of workers with repeat claims had changed both employers and workplace locations. The risk of a more rapid repeat claim was lowest in those workers who had changed both employers and workplaces. Similarly, those workers who had changed workplaces but retained the same employer had a significantly longer duration between initial and repeat claims than those that had remained at the same employer and workplace location (Table 5).

**Table 5:** Characterisation of repeat claims by workplace and employer categories.

Category*	N (%) of participants	**Mean ± SD days between 1**^**st **^**and 2**^**nd **^**claims**	HR [95% CI]
Same workplace location/Same employer *(Reference condition)*	34,626 (55.2%)	666 ± 757	1
Different workplace location/Same employer	6,508 (10.4%)	1,181 ± 1,031	.56 [.55 - .58]
Different workplace location/Different employer	21,626 (34.4%)	1,736 ± 1,139	.41 [.39 - .41]
Total	62,760	1,088 ± 1,057	

Mean ± SD days represents average time between the dates of claims lodged. Estimated Hazard Ratio (HR) from Cox regression with 95% Confidence intervals (CI) is shown in the right-most column.

* Workplace location and employer were identified from the jurisdictional compensation system database.

### Impact of repeat claims

The mean costs of the initial claim were significantly greater in Group 1 than Group 2 (Table 6). Total costs of the initial claim in Group 2 were 52.6% of those incurred for the initial claim in Group 1. Similarly, costs incurred for weekly compensation were 50.1% of those incurred in Group 1, while costs for medical and like services were 59.0% of those incurred in Group 1. The costs of the second claim in Group 2 were significantly greater than costs of the initial claim in the same group in all three cost categories. Total costs (or a total amount of all payments, including weekly payments, reasonable medical and like services, impairment benefits, common law damages, payments following a workplace death and superannuation entitlement) of repeat claims in this group were 149.1% higher than first claims, while weekly compensation and medical and like (rescue, medical, hospital, nursing, personal and household, occupational rehabilitation and ambulance) services were 185.2% and 142.5% higher, respectively. Some cost categories (total cost, medical and like services) of the repeat claim in Group 2 were significantly lower than those for the initial claim in Group 1. Total cost of repeat claims in Group 2 were 78.6% of those for the first claim in Group 1, while medical and like service costs were 83.7% of those for the first claim in Group 1.

**Table 6:** Indicators of claim impact in groups with single and repeat claims

	Group 1 (N = 107,388)	Group 2 (N = 62,760)		
Impact	Initial claim	Initial claim	Repeat claim	Sig*
Mean ± std total cost per claim	$16,731 ± $87,011	$8,813 ± $50,938	$13,147 ± $65,100	a,b,c
Mean ± std cost of weekly compensation paid	$5,249 ± $30,877	$2,631 ± $18,501	$4,875 ± $27,204	a,c
Mean ± std cost of medical and like services paid	$3,447 ± $29,128	$2,034 ± $9,996	$2,886 ± $13,025	a,b,c
Mean ± std days of incapacity for work	67 ± 278	42 ± 196	66 ± 244	a,c

* a = significant difference *(p < 0.001) *between Group 1 initial claim and Group 2 initial claim; b = significant difference between Group 1 initial claim and Group 2 repeat claim; c = significant difference between Group 2 initial claim and Group 2 repeat claim.

Work disability, as measured by the mean number of days that workers' had an incapacity for work (or days off work), was also significantly different between groups. The initial claim from Group 2 resulted in significantly fewer days of incapacity than both the initial claim from Group 1 and the second claim from Group 2. There was no difference between the initial claim from Group 1 and the repeat claim from Group 2.

## Discussion

This was a descriptive study of workers compensation claims from the state of Victoria in Australia. There has been relatively little attention paid to repeat claims in the published literature, and in our experience little attention has been paid to these claims by workers' compensation authorities. Thirty-seven percent of workers' with an accepted workers' compensation claim during the 5 year period of this study subsequently filed, and had accepted, a second claim (Table 1).

Repeat claimants were younger and more likely to be male than workers with a single claim. These findings are similar to self-report data collected from a large sample of recently injured workers who had filed workers' compensation claims in Australia, where 35% of injured workers reported having made a previous claim[14].

The majority of repeat claims were for a subsequent occupational injury, or an exacerbation of the existing occupational injury, both of which are permitted under the Victorian workers' compensation legislation. However, a substantial proportion (21.2%) of repeat claims was for occupational disease. The majority of second claims (61.4%) were for different afflictions than initial claims, and in most cases (65.8%) a different bodily location was involved. The same affliction to the same bodily location was noted in only 18.5% of repeat claims. Thus it appears that in the majority of cases the second claim was for a new occupational injury or disease.

The duration between first and second claims was significantly shorter in workers' whose circumstances had not changed between claims (eg, same employer/workplace, same occupation/industry). Those workers' who remained employed by the same employer at the same workplace location had the shortest duration (666 days or approximately 1 year & 10 months) between first and second claims, than those whose workplace location or employer had changed between first and second claims (Table 5). Similarly, workers' who remained employed in the same industry had a shorter duration between claims than those who had moved between industries (Table 4). In the present study, the period between initial and repeat claims is greater than that reported by Cherry and colleagues[12]. However, direct comparison is difficult given jurisdictional differences in eligibility for workers' compensation, benefit structures and occupational injury and disease prevention activities.

The costs and work disability arising from of an initial claim was significantly less in those workers' who subsequently filed a second claim. However, costs and work disability arising from repeat claims were much greater than the costs of initial claims in this group. Further investigation of potential reasons for this finding is required; however a number of possibilities are apparent. One factor influencing the likelihood of filing a repeat claim may be inappropriate or incomplete rehabilitation of the initial occupational injury. In the jurisdiction studied both exacerbations of existing injuries and new injuries can result in a new claim being filed. Alternatively, the repeat claim group included a substantial proportion of occupational disease claims. Work related disability and treatment costs associated with occupational disease may be greater than that associated with injury and this may have impacted our data.

Combined, the financial cost to the compensation authority of workers' with repeat claims was substantially greater than that incurred by workers' with only a single claim. The total average cost of compensating a worker with a repeat claim was $21,960 (for an initial and repeat claim) compared to $16,731 for a worker with a single claim.

Multiple claims also result in multiple periods of work incapacity. Inability to work has significant impacts on the individual, their family and community[15]. Thus there are both economic and social incentives for employers, health and safety agencies and workers' compensation authorities to target the prevention of subsequent injury and the exacerbation of existing injury among workers who have already filed and had accepted a compensation claim.

Of those workers' with a repeat claim, 65.6% were employed by the same employer at the time of both first and second claims. Thus, 24.3% (41,134) of the 170,148 workers whose initial claim was filed during the five year window of this study went on to file a second claim while working for the same employer. The duration between first and second claims in this group was also shorter than that in repeat claimants who had changed employers between claims. Further research is required to identify factors associated with this pattern of claiming behaviour. A number of factors may be at play, including those related to the employer (eg, safety policies and practices, return to work policies and practices), industry (eg, relative risk of injury or disease) or worker (eg, motivation to return to work, claiming behaviour). It could be that older workers may not file claims and they are less concerned about future employment because they are closer to retirement age or they have seniority and receive higher wages[16]. Another factor to consider is job tenure. Tompa and colleagues showed that job tenure and unionization were factors in sickness absence in workers with temporary employment[17]. The current results are also consistent with the suggestion that workers who have recently changed occupation, industry or employer may be less likely to file a workers compensation claim.

Limitations of the current dataset should be noted. First, it is well established that a proportion of work-related injuries and diseases do not appear on workers' compensation datasets[10]. Similarly, workers are less likely to claim compensation for psychological and social conditions than they are for conditions with a physical manifestation (eg, musculoskeletal disorders, traumatic injury)[18]. The decision to make a workers' compensation claim for a work-related condition may be influenced by a range of factors including the nature of the condition as well as its severity, jurisdictional eligibility and the workers awareness of entitlements[18]. Second, the workers' compensation authority from which this data was drawn does not have population coverage of work-related injuries in its jurisdiction. A small proportion (in the order of 10% to 15%) of workers are employed by organisations that self-insure or are covered under a federal workers' compensation agency, or are sole-traders and thus exempted from registering workers' compensation. Thus it is likely that a proportion of workers will have had workers' compensation provided by the Victorian state jurisdiction at the time of their initial injury but not at the time of the second injury. Conversely, a proportion of workers identified as 'single' claimants on our dataset may have previously filed workers' compensation claims in another jurisdiction, had work-related injuries that were not covered by workers' compensation, or have met eligibility criteria for compensation but not filed a claim. Thirdly, we do not have information on claimants return to work as these dates are not recorded consistently by the compensation authority, particularly for periods before the year 2004/5. The reliability of this data is improving as the VWA gains more experience with collecting return to work outcomes. In future we may be able to address this limitation. It is difficult to estimate the magnitude of these impacts without undertaking a comprehensive data linkage between jurisdictional workers' compensation and health datasets.

## Conclusions

The group of workers whose working conditions do not change between initial and repeat claims, and particularly that group who remain with the same employer, represent a unique prevention opportunity for government and industry. There is the potential to substantially reduce the social, health and economic burden of workplace injury by enacting prevention programs targeted at workers' who have already filed compensation claims. Both the injured worker and their employer are known to the workers' compensation authority. Prevention of subsequent workplace injury and disease in those workers' who have already filed a workers' compensation claim should be a focus for OH&S regulators and industry.

## Competing interests

Funding for this project and Dr Collie's involvement in the study was provided by a research grant from WorkSafe Victoria.

## Authors' contributions

Both authors equally participated in the study design and coordination. Both authors read and approved the final manuscript.

## Pre-publication history

The pre-publication history for this paper can be accessed here:

http://www.biomedcentral.com/1471-2458/11/492/prepub
